# Abattoir-based study on the epidemiology of caprine tuberculosis in Ethiopia using conventional and molecular tools

**DOI:** 10.1186/1751-0147-55-15

**Published:** 2013-02-21

**Authors:** Benti Deresa, Franz J Conraths, Gobena Ameni

**Affiliations:** 1 Aklilu Lemma Institute of Pathobiology, Addis Ababa University, PO Box 1176, Addis, Ethiopia; 2 Jimma University College of Agriculture and Veterinary Medicine, PO Box 307, Jimma, Ethiopia; 3 Friedrich-Loeffler Institute of Epidemiology, Seestraße 5516868, Wusterhausen, Germany; 4 Freie Universität Berlin, Koenigsweg 67, Berlin, 14163, Germany

**Keywords:** Goat, Tuberculosis, Epidemiology, Molecular typing, *Mycobacterium tuberculosis*, Ethiopia

## Abstract

**Background:**

Despite the important role of goats for meat and milk production in Ethiopia, little information is available on the epidemiology of caprine tuberculosis (TB). Caprine TB is important as milk is usually consumed raw particularly by Ethiopian pastoralists. The objectives of the present study were to estimate the prevalence of TB in goats at an abattoir, to evaluate associated risk factors and to characterize the causative mycobacteria.

**Methods:**

A cross-sectional study was conducted on 1990 randomly selected male goats that were slaughtered at Luna Export Abattoir of central Ethiopia. Postmortem examination, mycobacterial culturing and molecular typing techniques like genus typing, deletion typing and spoligotyping were used.

**Result:**

The overall prevalence of caprine TB-like lesions was 3.5%. The lesion prevalence increased significantly with increasing age. Mycobacteria were found by culture and seen as acid fast bacilli in 12% of the goats with TB-like lesions. Characterization of the eight isolates using multiplex polymerase chain reaction (PCR) indicated that five of them belonged to the genus *Mycobacterium*. Four of the latter were confirmed to be members of the *M. tuberculosis* complex. Further characterization of the three *M. tuberculosis* isolates by spoligotyping identified them as type SIT53 and two new spoligotypes.

**Conclusion:**

The isolation of *M. tuberculosis* from goats in this study indicates a potential risk of transmission of *M. tuberculosis* between humans and goats.

## Background

Caprine tuberculosis (TB) caused mainly by *Mycobacterium bovis* and *M. caprae*[1,2] poses a risk to goat health and production in developing world [3-5]. Recently, reports of caprine TB have increased in several countries; even in those practicing a long standing test and slaughter policy [2,3,5-10]. It is reported that the infection is widespread in Africa where goats co-graze with cattle that are not subject to TB testing and slaughter regimes [9-11]. Goats may become infected with *M. bovis* when sharing pastures with infected cattle, at watering points, market places and shared night shelters [6].

In Ethiopia, mixed farming of cattle and goats is a common practice. Livestock move freely from one region to another and from farm-to-farm. Thus, this practice poses a high risk of inter- and intraspecies transmission and spread of *M. bovis* infection [12]. This mixed farming of small and large ruminants is especially a risk to goats in countries like Ethiopia where bovine TB is endemic [13] and reports have shown a prevalence of bovine TB ranging from 3.4% in small holder production systems to 50% in intensive dairy production systems [14-17].

A previous study [18] reported a prevalence of 4.2% TB in Ethiopian goats and also reported the occurrence of the disease for the first time in Ethiopia. However, that study did not identify the species of the *M. tuberculosis* complex (MTBC). Thus, there is little information on the status and etiology of caprine TB in Ethiopia. As scientific knowledge is required to design appropriate control methods, this study was performed to estimate the prevalence of caprine TB in slaughter goats, evaluate associated risk factors, and characterize the causative agents.

## Materials and methods

### Study site and animals

This study was conducted in Luna Modern Export Abattoir at Modjo Town, which is located 73 km southeast of Addis Ababa in central Ethiopia. The abattoir is privately owned and primarily male goats are slaughtered for export purpose.

All goats were of local breeds and originated mainly from Arsi, Borana, Jimma, Somali and South Wello representing different agro-ecological zones. All goats had been kept under extensive production systems either as mixed crop–livestock production system or as a pastoral system of production. Goats were purchased at different local markets and transported to the abattoir. At the abattoir, animals were fed, watered, and rested for 24 to 72 hrs before being slaughtered. The TB status of the goats was not known as there was no pre-slaughter tuberculin skin testing scheme.

Our project was evaluated and approved by the Institional Review Board (IRB) of the Aklilu Lemma Institute of Pathobiology, Addis Ababa University. The Reference Number of the approval letter/Minutes is **IRB/01/2011-12**

### Study design and sampling techniques

A cross-sectional study design was used. The design involved stratification of the goats according to geographic origin and goat type to estimate the prevalence of caprine TB and assess the potential risk factors of the disease. The goat type was defined according to its origin and the physical description given earlier [4]. The study goats were also categorized into two age groups conventionally: ≤ 1.5 years as young, and >1.5 years as adult on the basis of the dentition as described earlier for African indigenous goats [19].

The selection of the goats was based on systematic random sampling while the goats were moving in line to the slaughter hall. The selected goats were identified using permanent marker, kept separately after selection and released for slaughter one after the other. The sampling interval was obtained by dividing the total number of animals slaughtered from specific geographical origin within specific day by the estimated daily sample size [20]. Twelve goats were sampled during every study day. Thus, the total number of animal slaughtered in the particular day from a particular origin was divided by twelve and every N^th^ animal was selected after random selection of the first animal until the daily sample size was met. Accordingly, a total of 1990 goats were examined.

### Postmortem examination

Postmortem inspection was performed following a procedure described previously [21]. All pulmonary lobes and the lymph nodes of the head (retropharyngeal, mandibular), thorax (mediastinal and bronchial), mesenterium, and liver were examined thoroughly. The carcass including internal organs and lymph nodes were examined under a bright-light source.

The lung and the lymph nodes were cut into approximately 2 cm thick slices to facilitate the detection of lesions using separate sterile scalpel blades. The cut surfaces were examined visually under bright light for the presence of lesions compatible with TB [22,23]. Gross nodular lesions of caseous necrosis and/or calcification were considered as suspected for TB. Such tissues were collected for bacteriological culture into sterile universal bottles with 5 ml of 0.9% saline solution. The samples were transported to Aklilu Lemma Institute of Pathobiology (ALIPB) on the same day and stored at +2 to +8°C for a maximum of one month until mycobacteriological culturing was carried out. However, almost all samples were cultured within a few days of sampling.

### Culturing and identification of mycobacteria

Specimen processing and culturing for mycobacteria was carried out at TB laboratory of ALIPB in accordance with the guidelines of the Office des Internationale Epizooties [24]. In the laboratory, individual tissue specimens were sectioned using sterile blades in sterile Petri dishes to obtain fine pieces and then homogenized with a mortar and pestle. The homogenate was decontaminated using 2 ml of 4% NaOH for 15 min and then centrifuged at 3,000 rpm for 15 min. The supernatant was discarded and the sediment was neutralized by 1% (0.1 N) HCl using phenol red as indicator. Neutralisation was considered to be achieved when the color of the solution was changed from purple to yellow.

Thereafter, 0.1 ml of the suspension was inoculated onto a duplicate set of Löwenstein-Jensen (LJ) slants; one supplemented with 0.4% sodium pyruvate (LJ pyruvate) and the other with glycerol (standard LJ). Cultures were incubated aerobically at 37°C for at least eight weeks with weekly observation for growth according to [25]. Positive cultures were confirmed by Ziehl–Neelsen staining and heat killed in water bath at 80°C for 45 min. The heat killed isolates was stored at −20°C for further molecular typing.

### Mycobacterial genus typing

Mycobacterial genus typing was done using polymerase chain reaction (PCR), which differentiates the MTBC from the *M. avium* complex, *M. intracellularae* and other mycobacterial species. PCR was conducted as described previously [26]. Heat killed Ziehl–Neelsen positive samples were used as source of the DNA template. DNA amplifications were done in a thermocycler with 20 μl reaction volume consisting: 5 μl of heat killed cells as a template, 8 μl HotstarTaqMasterMix (MgCL2, dNTP, Taq polymerase and PCR buffer) (Qiagen, United Kingdom) for each sample, 0.3 μl of each of the six primer per sample and 5.2 μl of Qiagen water per sample. The name and nucleotide sequence of each primer used for amplification in genus typing is given in Table 1.

**Table 1:** Primers used for genus and RD9 typing of isolated mycobacteria

**Primer name**	**Primer sequence(5**^′^** to 3**^′^**)**	**Product size**
MYCGEN-F	AGAGTTTGATCCTGGCTCAG	1030 bp
MYCGEN-R	TGCACACAGGCCACAAGGGA	
MYCAV-R	ACCAGAAGACATGCGTCTTG	180 bp
MYCINT-F	CCTTTAGGCGCATGATGTCTTTA	850 bp
TB1-F	GAACAATCCGGAGTTGACAA	372 bp
TB-R	AGCACGCTGTCAATCATGTA	
RD9_IntR	CTGGACCTCGATGACCACTC	396 bp (presence of RD9)
RD9_FlankF	AACACGGTCACGTTGTCGTG	575 bp (absence of RD9)
RD9_FlankR	CAAACCAGCAGCTGTCGTTG	

*M. tuberculosis* strains (H37Rv) and *M. avium* were used as positive controls while Qiagen water was used as negative control. The reaction mixture was then heated in a Programme Thermal Controller (Applied Biosystem; PTC- 100™) using the following amplification programs: 95°C for 10 min for enzyme activation; 95°C for 1 min for denaturation; 61°C for 0.5 min for annealing; 72°C for 2 min for extension; involving 35 cycles all in all and final extension at 72°C for 10 min.

A 1.5% agarose gel was prepared and the products were electrophoresed in 10×TAE running buffer. Ethidium bromide at ratio of 1:10, 100 bp DNA ladder, and orange 6x loading dye were used in gel electrophoresis. Finally, bands were visualized using alpha innotech, version 1.2.0.1 (Alpha Innotech Corporation) in a multi–image™ light cabinet.

### Region of difference (RD) deletion typing

For deletion typing, the procedure described earlier [16] was followed. The RD9 deletion typing was carried out on isolates that showed band for *M. tuberculosis* complex by genus typing. Each sample was tested in a separate PCR tube. Primers directed against the RD9 were used to generate a deletion profile that would allow species identification of the isolate.

RD9 is a 2030 base pair (bp) gene segment of *M. tuberculosis* and PCR analysis using flanking primers revealed that RD9 is absent in *M. bovis*, *M. microti*, and *M. africanum*[27]. Primers used for RD9 typing and the size of PCR product size expected in the presence or absence of the respective region of difference is given in Table 1.

The reaction mixture consisted of: 10 μl of HotStarTaq master mix, 0.3 μl × 3 of each primer (flank_R, F and Int) of the respective deletion typing, 2 μl DNA template and 7.1 μl Qiagen water to a final volume of 20 μl. *M. tuberculosis* H37Rv and *M. bovis* 2122/97 were used as positive controls while Qiagen water was used as negative control. The mixture was heated in a Programme Thermal Controller (Applied Biosystem; PTC- 100™) using an initial hot start of 95°C for 15 min followed by 35 cycles of 95°C for 1 min; 55°C for 1 min; and 72°C for 1 min; a final extension step of 72°C for 10 min to complete the cycle. PCR products were electrophoresed in 1.5% agarose gel in 10× TAE running buffer. Ethidium bromide at ratio of 1:10,100 bp DNA ladder and orange 6× loading dye (Gene Craft, Germany) were used in electrophoresis. Finally, bands were visualized using alpha innotech, version 1.2.0.1 (Alpha Innotech Corporation) in a multi–image™ light cabinet.

### Spoligotyping

Spoligotyping was performed at ALIPB; following the procedure described earlier [28] and according to the spoligotype kit supplier’s instructions (Ocimum Biosolutions Company, Iisselstein, The Netherlands). The direct repeat (DR) region were amplified by PCR using oligonucleotide primers derived from the DR sequence (DRa:5^′^-GGT TTT GGG TCT GAC GAC-3^′^ and DRb:5^′^-CCG AGA GGG GAC,GGA AAC-3^′^). A total volume of 25 μl of the following reaction mixture was used for the PCR: 12.5 μl of HotStarTaq master mix (Qiagen). This solution provides a final concentration of: 1.5 μM MgCl2 and 200 mM of each deoxynucleotides triphosphates, 2 μl of each primer (20 p mol each), 5 μl suspension of heat-killed cells (approximately 10 to 50 ng), and 3.5 μl distilled water. PCR amplification was performed for 15 min at 96°C and then subjected to 30 cycles of 1 min at 96°C; 1 min at 55°C, 30 sec at 72°C and a final extension at 72°C for 10 min.

The amplified products were hybridized to a set of 43 immobilized oligonucleotides, each corresponding to one of the unique spacer DNA sequences within the DR locus. After hybridization, the membrane was washed twice for 10 min in 2× SSPE (1× SSPE is 0.18 M NaCl, 10 μM NaH2PO4, and 1 μM EDTA (pH 7.7) - 0.5% sodium dodecyl sulfate at 60°C and then incubated in 1:4,000 diluted streptavidin peroxidase (Boehringer, Ingelheim Germany) for 45 to 60 min at 42°C. The membrane was washed twice for 10 min in 2× SSPE - 0.5% sodium dodecyl sulfates at 42°C and rinsed with 2× SSPE for 5 min at room temperature 20°C). Hybridizing DNA (presence or absence of the unique spacers) were detected by the enhanced chemiluminescence method (Amersham, Buckinghamshire, England) and by exposure to X-ray film (Hyperfilm ECL, Amersham), which detects light signals and thereby produces a pattern which allows for typing of isolates as specified by the manufacturer.

### Data collection, management and analysis

For each individual animal examined age, goat type and geographical origin were recorded on a data sheet. Presence or absence of TB-like lesions and affected tissue(s) were recorded in database based on Microsoft® Excel for Windows 2007.

Descriptive statistics was used to estimate prevalence of TB-like lesions across the individual factors and lesion frequency in different anatomical locations. Uni-and multivariate logistic regressions were used to investigate possible associations between the prevalence and the explanatory variables. *P* value <0.05 and 95% confidence level were used for statistical significance. Statistical analysis was carried out using SPSS version 18.0 (SPSS Inc. Chicago, IL, USA).

## Results

### Prevalence of TB-like lesions in goats and associated risk factors

The prevalence of TB-like lesions was 3.5% (2.69-4.31%). It was significantly higher in older goats (>1.5 year) than in younger goats (*P* < 0.05) but there was no statistically significant difference in lesion prevalence between the different goat types and origins (*P* > 0.05) (Tables 2 and 3). The highest (4.9%) and lowest (2.1%) prevalences of caprine TB-like lesions were recorded in goats originated from Borena and Somali areas, respectively.

**Table 2:** Individual variables, prevalence, and univariate analysis of risk factor of tuberculosis-like lesions in slaughtered goats

**Variables**	**No of carcasses examined**	**No (% positive)**	**χ**^**2 **^**–value**	***P*-value**
**Age**			6.436	0.011*
≤1.5 year	1048	26(2.5)		
>1.5 year	942	43(4.6)		
**Origin**			6.140	0.189*
Borena	472	23 (4.9)		
Arsi	360	13 (3.6)		
Jimma	386	15 (3.9)		
South wello	386	10 (2.6)		
Somali	386	8(2.1)		
**Goat type**			1.165	0.76(NS**)**
Somali	858	31(4.5)		
Arsi-Bale	360	13 (3.6)		
C. Lowlands	386	15 (3.9)		
Afar	386	10 (2.6)		

*-the value is significant; NS- not significant.

**Table 3:** Multivariate analysis of risk factor with presence of tuberculosis-like lesions in goats

**Variable**	**No. of carcasses examined**	**OR**	***P*-value**	**95% CI**
**Age of goat**				1.142 - 3.096
**≤**1.5 year	1048	1	1	
>1.5 year	942	1.88	0.013*	
**Origin of goat**				
Somali	386	1	1	
Borena	472	2.14	0.034*	1.068 - 5.473
Arsi	360	1.83	0.187 (NS)	0.746 - 4.462
Jimma	386	1.78	0.196 (NS)	0.744 - 4.258
South Wello	386	1.24	0.65 (NS)	0.485 - 3.187

*-the value is significant; NS- not significant.

### Distribution of pathology

**Figure 1:**
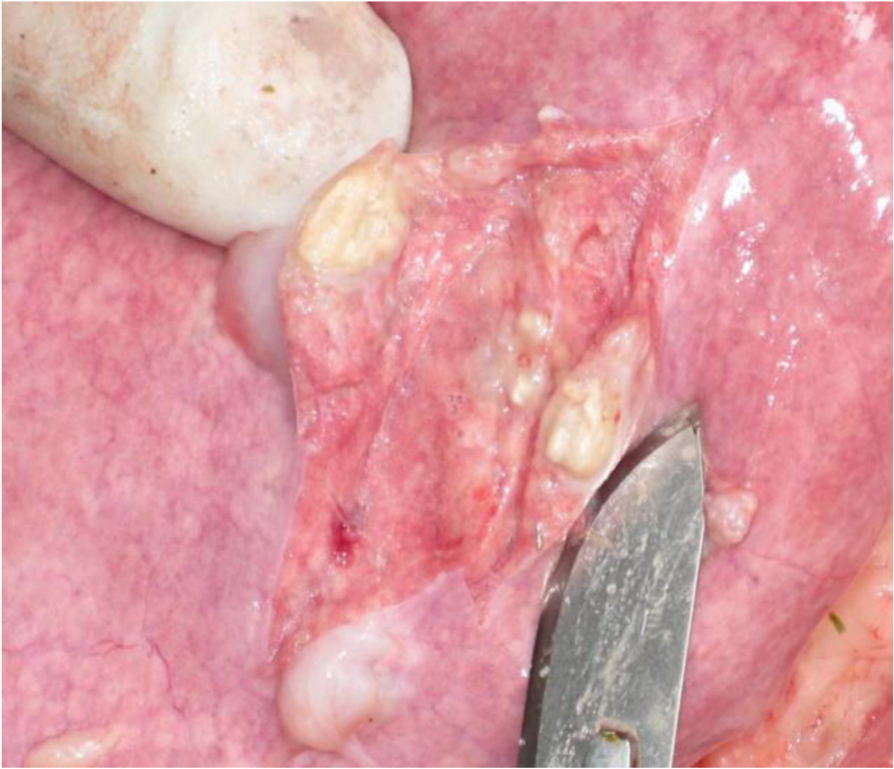
Irregularly shaped, soft white foci of granulomatous inflammation (TB-like lesion) on the pleural and cut surfaces of a goat lung.

Characteristic TB-like lesions (Figure 1) were observed in both lung and lymph nodes.

Most of the TB-like lesion were observed in the thoracic cavity (lung: 22% of cases, mediastinal lymph nodes: 42%, and bronchial lymph nodes: 33%) and only 3% was observed in mesenteric and hepatic lymph nodes.

### Culture and acid fast test microscopy results

Seventy-eight samples from suspected TB lesions were processed and cultured. Bacterial growth was observed in 14% (11/78) of the sowed slants of which eight were confirmed to be acid fast bacilli (AFB).

### Genus identification of AFB isolates

**Figure 2:**
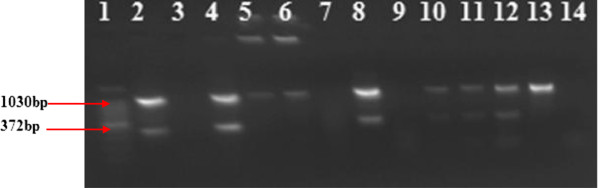
**Gel Electrophoretic separation of PCR products by multiplex PCR genus typing of mycobacterial isolate from tuberculous tissue of goats.** Lanes: 1 = 100 bp DNA Ladder, 2 = *M. tuberculosis* (positive control), 3 = Qiagen H2O (negative control), 4 = *M. bovis,* 5 = *M. av*ium complex (positive control) and 6 = *M. intracellulare* complex (positive control). Lanes 7–14 were samples from individual goats with TB-like lesions. Lanes 8, 10,11,12,13 were positive for the Genus *Mycobacterium* Lanes 8 and 10–12 showed bands for *M. tuberculosis* complex (MTBC) and lane 13 was positive only for the Genus *Mycobacterium* while lanes 7, 9 and 14 were negative.

Genus typing revealed that five of the eight AFB positive isolates showed the expected PCR product (1030 bp) and could be identified as *Mycobacterium* species. Furthermore, four of these five isolates generated a PCR product of 372 bp which belongs to MTBC group whereas the remaining isolate was considered to be atypical mycobacteria (Figure 2).

### RD9 deletion typing result

**Figure 3:**
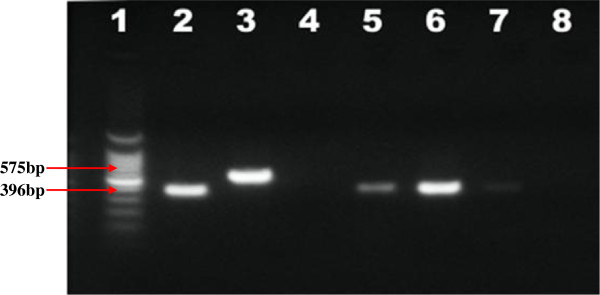
**Electrophoretic separation of PCR products of RD9 deletion typing.** Lanes: 1 = 100 bp DNA Ladder, 2 = *M. tuberculosis* (positive control), 3 = *M. bovis* (positive control), 4 = Qiagen H2O (negative control), Lanes 5–7 were isolates which generated a PCR product of 372 bp in Genus typing.

To identify the species of the MTBC isolates, RD9 deletion typing was performed. In this deletion typing, all the three isolates generated a PCR product of 339 bp confirming that they were *M. tuberculosis* (Figure 3) while one of the samples (Lane 8) did not produce the band (negative).

### Spoligotyping of M. tuberculosis isolates

**Figure 4:**
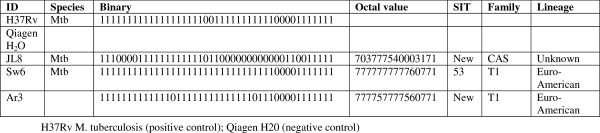
**Spoligotype patterns of *M. tuberculosis *isolates from goats.** One of the goat isolate was SIT53 while the other two were new to the Spoligotype International Typing (SITVIT) Database. The octal numbers of the two new strains are 703777540003171 and 777757777560771.

All the three isolates of *M. tuberculosis* showed distinct patterns indicating that they were different strains (Figure 4). One of these strains (SW6) was SIT53. However, the patterns of the remaining two strains (Ar3 and Jl8) were not recognized by the international spoliotyping database indicating that they were new strains.

## Discussion

An abattoir-based epidemiological survey of caprine TB was conducted on goats originated from different regions of Ethiopia. Lesion-based prevalence was calculated and mycobacteria were characterized. Compared to the two previous studies carried out on goat TB in Ethiopia, this study was more comprehensive as the number of goats included was relatively higher, the origin and types of animals was more representatives of the reference population and advanced molecular techniques were used.

The overall lesion prevalence of 3.5% reported in this study is similar to the prevalence reported in previous studies in Ethiopia. A TB-like lesion prevalence of 4.2% in 1,536 goats was reported in a study conducted in 2005 at Modjo Modem Export Abattoir [18]. A similar prevalence (4.3% in 1,152 goats) was recorded based on gross lesions at Helmex Export Abattoir [10]. Likewise, a prevalence of 3.1% in 193 goats was reported in goats kept at Adami Tulu Research Center using a single intradermal tuberculin test (TST) [29]. Studies conducted in other countries have also reported similar results [3,30].

A significantly higher prevalence of TB-like lesions in goats aged >1.5 years is in agreement with a study [30], which reported higher prevalence in older goats although the goats were older than 6 years in that study. Similarly, several cross sectional studies conducted on bovine TB have reported higher prevalences of TB in older cattle [11,14,31]. This may be explained by an age depend risk of exposure.

The prevalences of TB in goats that originated from the different zones and regions were similar indicating the overall similarity of management system and infection pressure. This finding is consistent with that of an earlier study [6], which reported similar prevalences in different regions of Ethiopia. A significantly higher prevalence was recorded for goats originating from the Borena area as compared to those from Somali region although the goats types were the same in both areas.

The present study showed that TB-like lesions were almost exclusively observed in the thoracic cavity of goats. A similar study has shown that 97% of the gross TB-like lesions were found in the lungs and associated lymph nodes [18], while lesions were only found in the thoracic cavity in Algerian goats [6]. This indicates that goats acquire the infection mainly through the respiratory route [32].

The isolation of three *M. tuberculosis* strains was unexpected as a previous study has reported that the causative agent of caprine TB in some European countries was *M. bovis*[9,11] and *M. caprae* in several European countries [33]. Even recently, outbreaks of TB in goats in the United Kingdom, Italy and Portugal were reported to be caused by *M*. *bovis*[2,5,34]. However, *M. tuberculosis* has previously been isolated from goats in Nigeria [3]. The isolation of *M. tuberculosis* from goats in this study is likely to be due to transmission of the bacterium from TB infected people to goats as has been suggested earlier [11]. Transmission of *M. tuberculosis* from man to cattle has been reported from Slovenia [35] and in Ethiopia; cattle owned by farmers with active TB were four times more likely to have TB than cattle owned by those farmers without active TB [7].

## Conclusion

This study documented an equal low prevalence of caprine TB-like lesions across a range of eco-epidemiological settings in Ethiopia. The high prevalence of lesions in the lung and associated lymph nodes suggests that transmission of TB to goats is mainly through the respiratory route. The isolation of the SIT53 strain of *M. tuberculosis* from goats in this study suggests transmission from humans.

## Competing interests

We have no financial or other competing interests to declare in relation to this manuscript.

## Authors’ contributions

The study was conceived and designed by BD, GA and FJC. Field work was performed by BD; Laboratory work was done by BD and GA. Analysis of data and preparation of the manuscript was a joint contribution of BD, FJC and GA. All authors read and approved the final version of the manuscript.

## References

[B1] Aranaz A Liebana E Gomez-Mampaso E Galan JC Cousins D Ortega A Blazquez J Baquero F Mateos A Suarez G Dominguez L *Mycobacterium tuberculosis* subsp. *caprae* subsp. *nov:***A taxonomic study of a new member of the***Mycobacterium tuberculosis* complex isolated from goats in Spain. Int J Sys Bacteriol 1999 49 1263 1273 10.1099/00207713-49-3-1263 10425790

[B2] Daniel R Evans H Rolfe S Outbreak of tuberculosis caused by *Mycobacterium bovis* in golden Guernsey goats in Great Britain Vet Rec 2009 165 335 342 10.1136/vr.165.12.335 19767636

[B3] Cadamus S Adesokan H Jenkins A Van Solingen D *Mycobacterium tuberculosis* and *M. bovis* in goats, Nigeria Em Inf Dis 2009 15 2066 2069 10.3201/eid1512.090319 PMC3044523 19961707

[B4] Hiko A Agga GE First-time detection of mycobacterium species from goats in Ethiopia Trop Anim Hlth Prod 2010 43 133 139 10.1007/s11250-010-9665-4 20725858

[B5] Tafess K Dawo F Sori T Ameni G Prevalence of caprine tuberculosis in Mid-Rift Valley area of Oromia, Ethiopia Afr J Microbio Res 2011 5 1473 1478

[B6] Naima S Borna M Bakir M Djamel Y Fadila B Jacob Z Djamel G Tuberculosis in cattle and goats in the North of Algeria Vet Res 2011 4 100 103

[B7] Regassa A Medhin G Ameni G Bovine tuberculosis is more prevalent in cattle owned by farmers with active tuberculosis in central Ethiopia Vet J 2008 178 119 125 10.1016/j.tvjl.2007.06.019 17761442

[B8] Sharpe AE Brady CP Johnson AJ Byrne W Kenny K Costello E Concurrent outbreak of tuberculosis and caseous lymphadenitis in a goat herd Vet Rec 2010 166 591 592 10.1136/vr.b4825 20453237

[B9] Prodinger WM Brandstätter A Naumann L Pacciarini M Kubica T Boschiroli ML Aranaz A Nagy G Cvetnic Z Ocepek M Skrypnyk A Erler W Niemann S Pavlik I Moser I Characterization of *Mycobacterium caprae* isolates from Europe by mycobacterium interspersed repetitive unit genotyping J Clin Microbiol 2005 43 4984 4992 10.1128/JCM.43.10.4984-4992.2005 16207952 PMC1248478

[B10] Nigussie T Preliminary study of tuberculosis in small ruminants slaughtered at helimex export abattoir 2005 Debre-Zeit, Ethiopia: DVM thesis Faculty of Veterinary Medicine

[B11] O’Reilly LM Daborn CJ The epidemiology of *Mycobacterium bovis* infections in animals and man: a review Tuber Lung Dis 1995 76 1 46 7579326 10.1016/0962-8479(95)90591-x

[B12] Ameni G Desta F Firdessa R Molecular typing of *Mycobacterium bovis* isolated from tuberculosis lesions of cattle in north eastern Ethiopia Vet Rec 2010 167 138 141 10.1136/vr.b4881 20656993

[B13] Hailemariam S A brief analysis of activities of meat inspection and quarantine division 1975 Addis Ababa, Ethiopia: Department of Veterinary Service, Ministry of Agriculture

[B14] Ameni G Aseffa A Engers H Young D Gordon S Hewinson G Vordemieier M High prevalence and increased severity of pathology of bovine tuberculosis in Holsteins compared to zebu breeds under field cattle husbandry in central Ethiopia ClinVac Immunol 2007 14 1356 1361 10.1128/CVI.00205-07 PMC2168113 17761523

[B15] Ameni G Ragassa A Kassa T Medhin G Survey on bovine tuberculosis and its public health implications to cattle raising families in Wolaita Soddo, Southern Ethiopia Ethiop J. Anim Prod 2001 1 55 62

[B16] Berg S Firdessa R Habtamu M Gadisa E Mengistu A Yamuah L Ameni G Vordermeier M Robertson BD Smith NH Engers H Young D Hewinson RG Aseffa A Gordon SV The burden of mycobacterial disease in Ethiopian cattle: implication for public health PLoS One 2009 4 e5068 e5070 10.1371/journal.pone.0005068 19352493 PMC2662418

[B17] Tsegaye W Aseffa A Machec A Mengist Y Bergd S Ameni G Conventional and molecular epidemiology of bovine tuberculosis in dairy farms in Addis Ababa City, the capital of Ethiopia Int J Appl Res Vet Med 2010 8 143 151

[B18] International Livestock Research Institute (ILRI) Goat types of Ethiopia and Eritrea: physical description and management system 1996 Nairobi, Kenya: FARM-Africa

[B19] Steele M Goats Tropical Agriculturist and Technical Center for Agricultural and Rural Co-operation 1996 London: Macmillan education Ltd 120 125

[B20] Thrusfield M Survey Veterinary Epidemiology 2005 Blackwell Science Ltd 228 242

[B21] Corner LA Post-mortem diagnosis of *Mycobacterium bovis* infection in cattle Vet Microbiol 1994 40 53 63 10.1016/0378-1135(94)90046-9 8073629

[B22] Asseged B Woldesenbet Z Yimer E Lemma E Evaluation of abattoir inspection for the diagnosis of *M. bovis* infection in cattle at Addis Ababa Abattoir Trop Anim Hlth Prod 2004 36 537 546 10.1023/b:trop.0000040934.32330.44 15560514

[B23] Gracy JF Meat Hygiene 1986 London: Bailliere-Tindall

[B24] OIE Bovine tuberculosis Manual of Diagnostic Tests and Vaccines for Terrestrial Part 2, Section 2.3. Chapter 2.3.3 2010 World Organization for Animal Health http://www.oie.int/international-standard…/terrestrial-manual/access-online

[B25] McIlroy SG Neill SD McCracken RM Pulmonary lesions and *Mycobacterium. bovis* excretion from the respiratory tract of tuberculin reacting cattle Vet Rec 1986 118 718 721 10.1136/vr.118.26.718 3526702

[B26] Wilton S Cousins D Detection and identification of multiple *mycobacterial* pathogens by DNA amplification in a single tube PCR Methods Appl 1992 1 269 273 10.1101/gr.1.4.269 1282431

[B27] Gordon SV Brosch R Billaut T Garnier K Eiglmeier Cole ST Identification of variable regions in the genomes of tubercle bacilli using bacterial artificial chromosome arrays Mol Microbiol 1999 32 643 655 10.1046/j.1365-2958.1999.01383.x 10320585

[B28] Kamerbeek J Schouls L Kolk A Van Agterveld M Van Soolingen D Kuijper S Bunschoten A Molhuizen H Shaw R Goyal M Van Embden J Simultaneous detection and strain differentiation of *Mycobacterium tuberculosis* for diagnosis and epidemiology J Clin Microbiol 1997 35 907 914 9157152 10.1128/jcm.35.4.907-914.1997 PMC229700

[B29] Crawshaw T Daniel R Clifton-Hadley R Clark J Evans H Rolfe S de la Rua Domenech R TB in goats caused by *Mycobacterium bovis* Vet Rec 2008 163 127 132 18660526 10.1136/vr.163.4.127

[B30] Javed MT Ahmad L Irfan M Ali I Khan A Wasiq M Farooqi FA Latif MS Cagiola M Hematological and serum protein values in tuberculin reactor and non-reactor water buffaloes, cattle, sheep and goats Pak Vet J 2010 30 100 104

[B31] Biffa D Inangolet F Oloya J Asseged B Badaso M Yilkal A Skjerve E Prevalence of bovine tuberculosis in Ethiopian slaughter cattle based on post-mortem examination Trop Anim Hlth Prod 2009 41 755 765 10.1007/s11250-008-9248-9 19058024

[B32] Vordermeier HM Chambers MA Cockle PJ Whelan AO Simmons J Hewinson RG Correlation of ESAT-6-specific gamma interferon with pathology in cattle following *Mycobacterium bovis* BCG vaccination against experimental bovine tuberculosis Infect Immunol 2002 70 3026 3032 10.1128/IAI.70.6.3026-3032.2002 12010994 PMC128013

[B33] Ayele WY Neill SD Zinsstag J Pavlik I Bovine tuberculosis: an old disease but a new threat to Africa Int J Tub Lung Dis 2004 8 924 937 15305473

[B34] Quintas H Reis J Pires I Alegria N Tuberculosis in goats Vet Rec 2010 166 437 438 20364017 10.1136/vr.c1678

[B35] Ocepek M Pate M Olnir-Dovc M Poljak M Transmission of *Mycobacterium tuberculosis* from human to cattle J Clin Microbiol 2005 43 3555 3557 10.1128/JCM.43.7.3555-3557.2005 16000505 PMC1169140

